# Supplementary material for: Tuning the Immunostimulation Properties of Cationic Lipid Nanocarriers for Nucleic Acid Delivery
Source: Front Immunol. 2021 Aug 23;12:722411. doi: 10.3389/fimmu.2021.722411 (PMC8419413; doi:10.3389/fimmu.2021.722411)
Supplement: Supplementary file 1 [file DataSheet_1.docx]

Supplementary Material

## Supplementary Figures 1.

|  | nNLCs | cNLCs |
| --- | --- | --- |
| Hydrodynamic diameter (nm) | 52.68 ± 0.15 | 45.18 ± 0.35 |
| PdI | 0.12 ± 0.01 | 0.23 ± 0.007 |
| Zeta potential (mV) | -16.5 ± 0.53 | 45.8 ± 3.8 |

The hydrodynamic diameter and PDI were measured with a dispersion of 1 mg/mL NLCs in PBS while the zeta potential was measured with a dispersion of 1 mg/mL NLCs in 1 mM NaCl. Each assay was performed in three replications at 25°C.


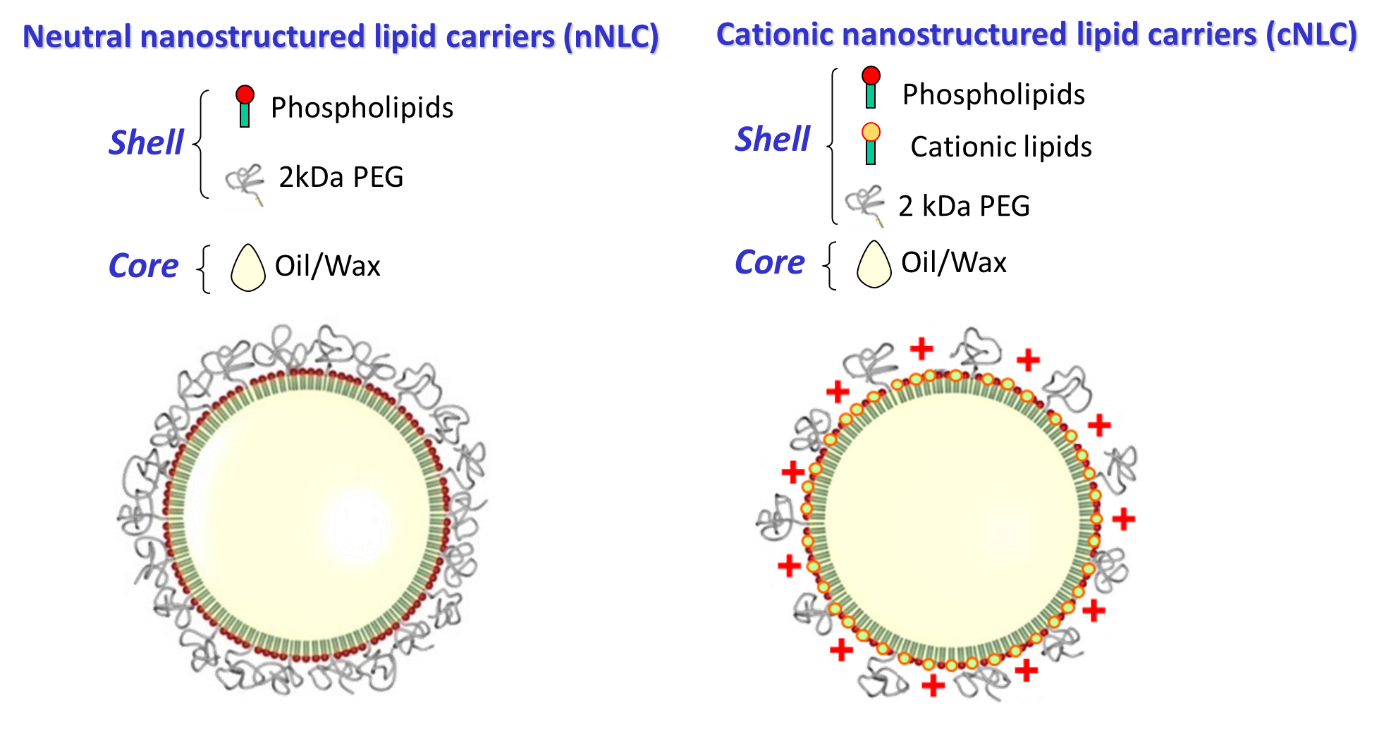


Supplementary Figure 1 | Physical characterization of the NLCs

Comment: The NLCs particles are made of a core with soybean oil and wax surrounded by a monolayer of phospholipids and a dense PEGylated coating with 2 kDa stearate PEG chains. This composition and the manufacturing process leads to the formation of very small stabilized lipid droplets, for further information, please see Delmas et al, Langmuir 2011, 27(5), 1683–1692. These particles and their cationic counterparts are therefore smaller from the cationic lipid particles described in the literature, including SLN and NLCs (Eramus et al, Molecular Therapy Volume 26, Issue 10, 3 October 2018, Pages 2507-2522 showing studies with 90 nm-diameter NLCs; Brito et al, Mol. Ther., 22 (2014), pp. 2118-2129; cationic emulsion of 100-130 nm).

## Supplementary Figures 2.


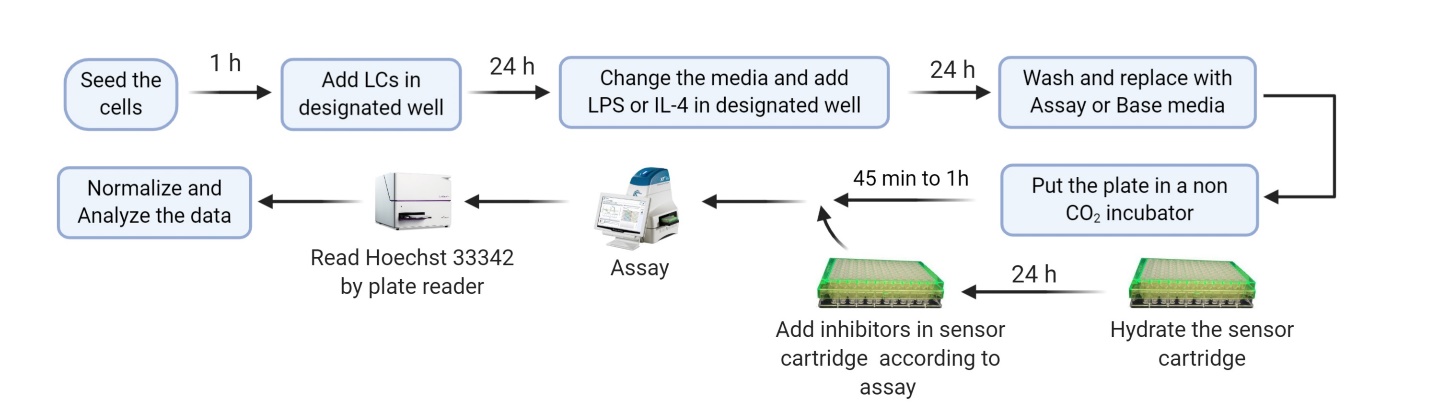


Supplementary Figure 2 | Experimental design of metabolic flux analysis

Mature BMDCs and BMDMs were seeded on a Seahorse culture plate. One hour after plating, cells were treated with the different nanocarriers. After 24 h of culture, cells were washed and when indicated, stimulated with LPS or IL-4 for 24 h. The metabolic analysis was performed using a Seahorse bio-analyser using the Mito Stress and Glyco Stress assay protocol, with the corresponding chemical inhibitors.

## Supplementary Figures 3


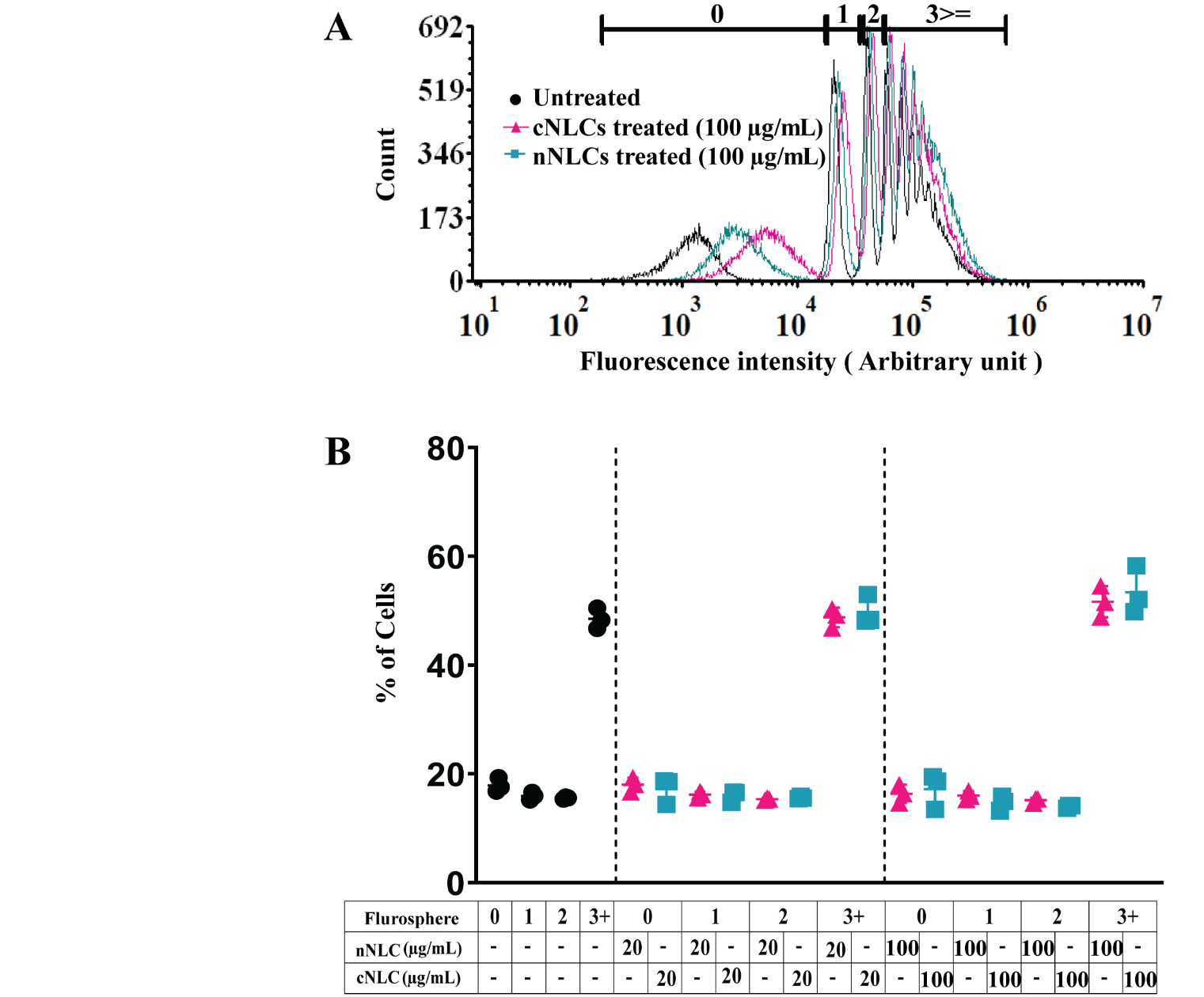


Supplementary Figure 3 | Phagocytosis capacity of macrophage cell line J774.1A

J774.1A cells were exposed to nNLCs and cNLCs nanocarriers at 100 µg/mL for 24 h, then incubated with fluorescent microspheres for 6 h, and subsequently analysed by flow cytometry. The repartition of the cells in the 1st, 2nd 3rd and 4th peak corresponds to 0, 1, 2 and 3 or more beads internalization, respectively. Overlaid histograms are shown in **(A)** The proportion of cells in each peak was analysed **(B)**. Data is displayed as mean ± SD (N = 3 independent experiments).

## Supplementary Figures 4


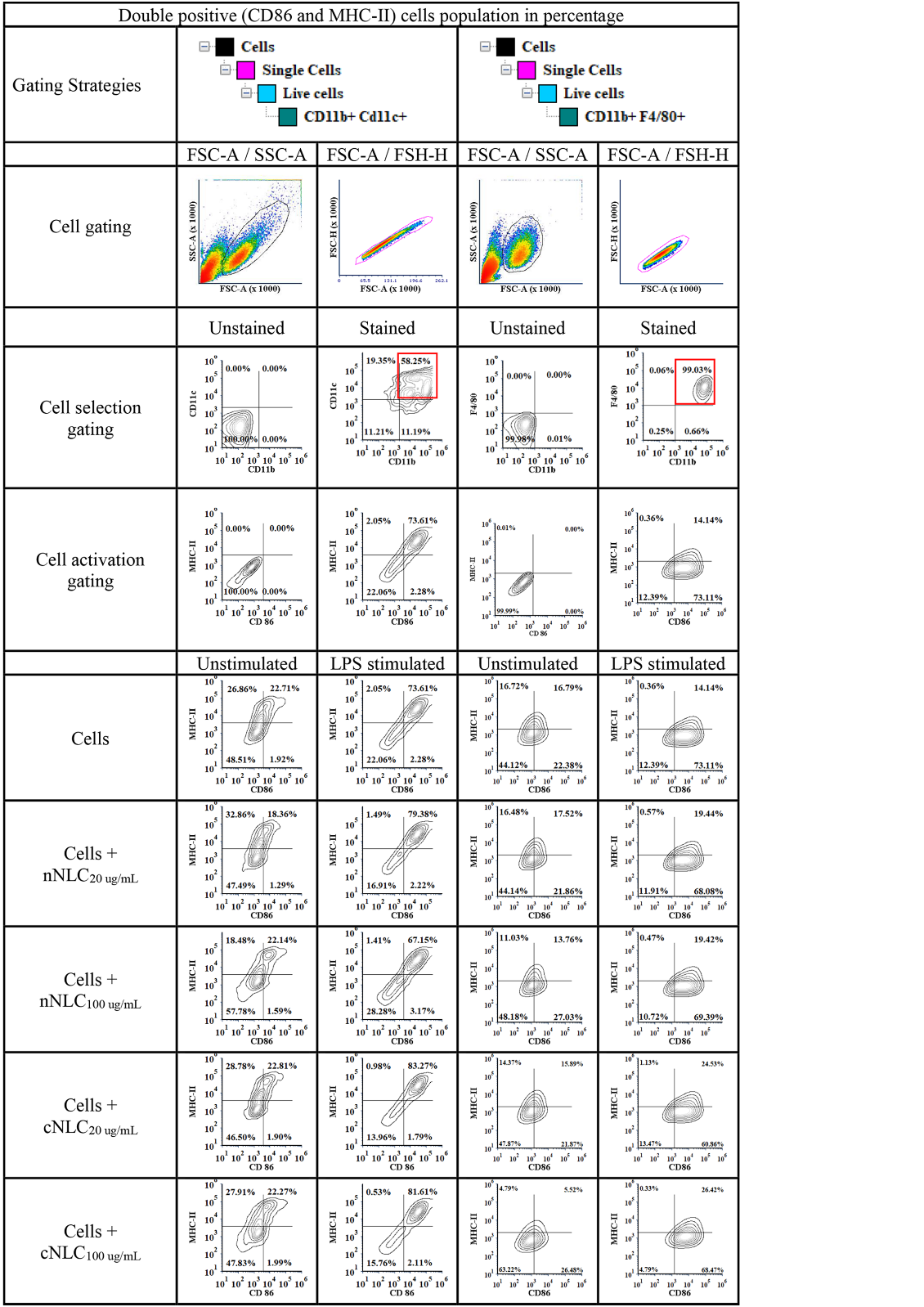


Supplementary Figure 4 | Expression of activation surface marker in APCs

The expression of activation marker for BMDCs and BMDMs was quantified by flow cytometry after exposure to nNLCs or cNLCs for 24 h, followed by LPS stimulation for additional 24 h when indicated. The percentage of double positive (CD86 and MHC-II) BMDCs and BMDMs were gated on CD11b and Cd11c positive cells for BMDCs and CD11b and F4/80 positive cells for BMDMs and contour graph was displayed. The results are representative one of the three independent experiment.

## Supplementary Figures 5.


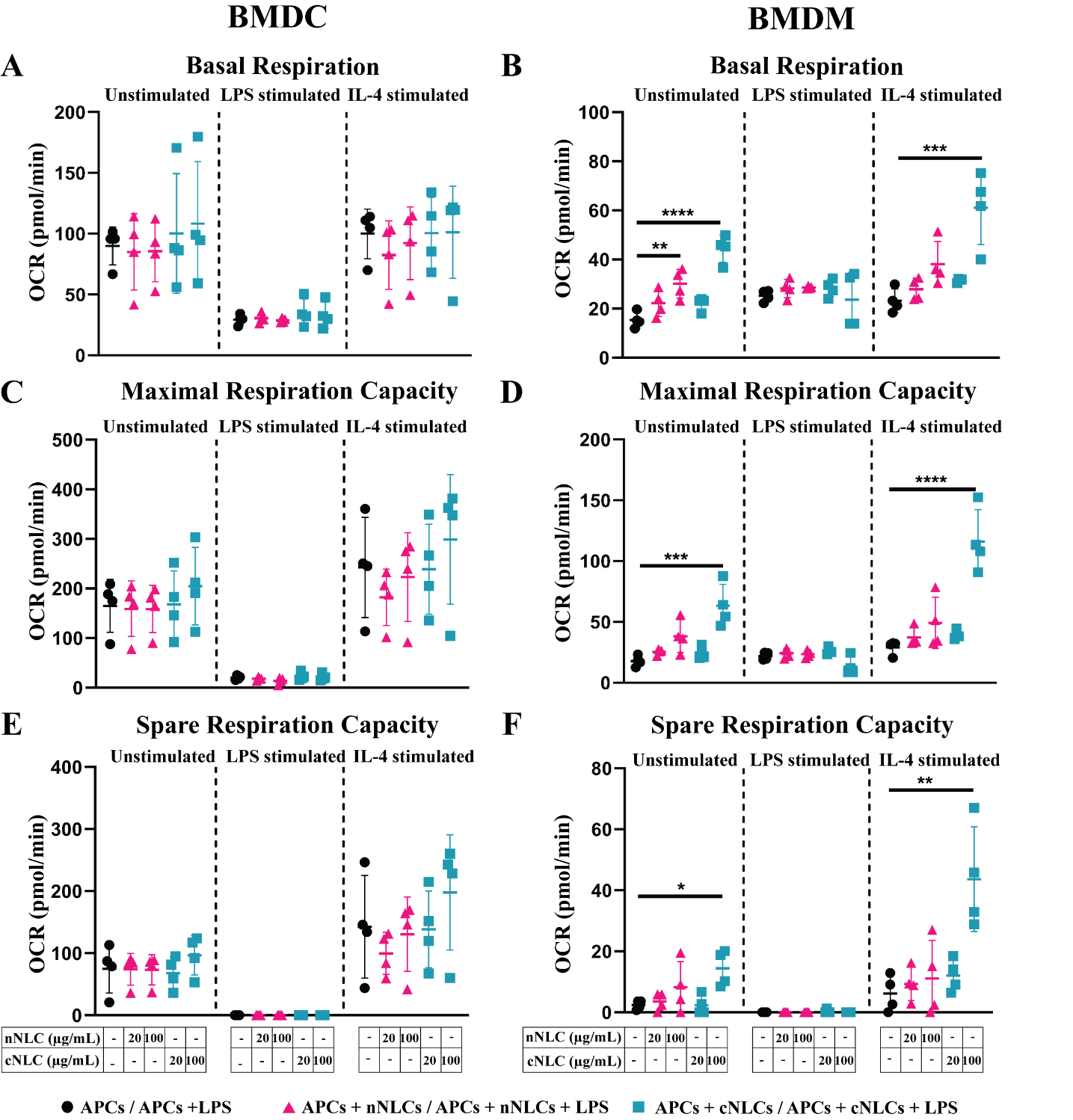


Supplementary Figure 5 | Basal respiration, maximal respiration capacity, and spare respiratory capacity of naïve, classically activated or alternatively activated APCs in response to nNLCs or cNLCs. **(A, B)** Basal respiration, **(C, D)** Maximal respiration capacity, **(E, F)** Spare respiratory capacity of BMDCs and BMDMs respectively were measured after exposure to cNLCs or nNLCs for 24 h and activated by LPS or IL-4 for another 24 h. Oxygen consumption rate (OCR) was quantified using a Seahorse XF analyser. Data was normalized by cell number based on cell count (Hoechst 33342 staining) and is displayed as mean ± SD (N = 4 independent experiments). Statistical significance between nanocarrier treated or untreated groups was performed by one-way ANOVA test using Tukey's multiple comparisons test. *P ≤ 0.05; **P ≤ 0.01; ***P ≤ 0.001; ****P ≤ 0.0001.

## Supplementary Figures 6.


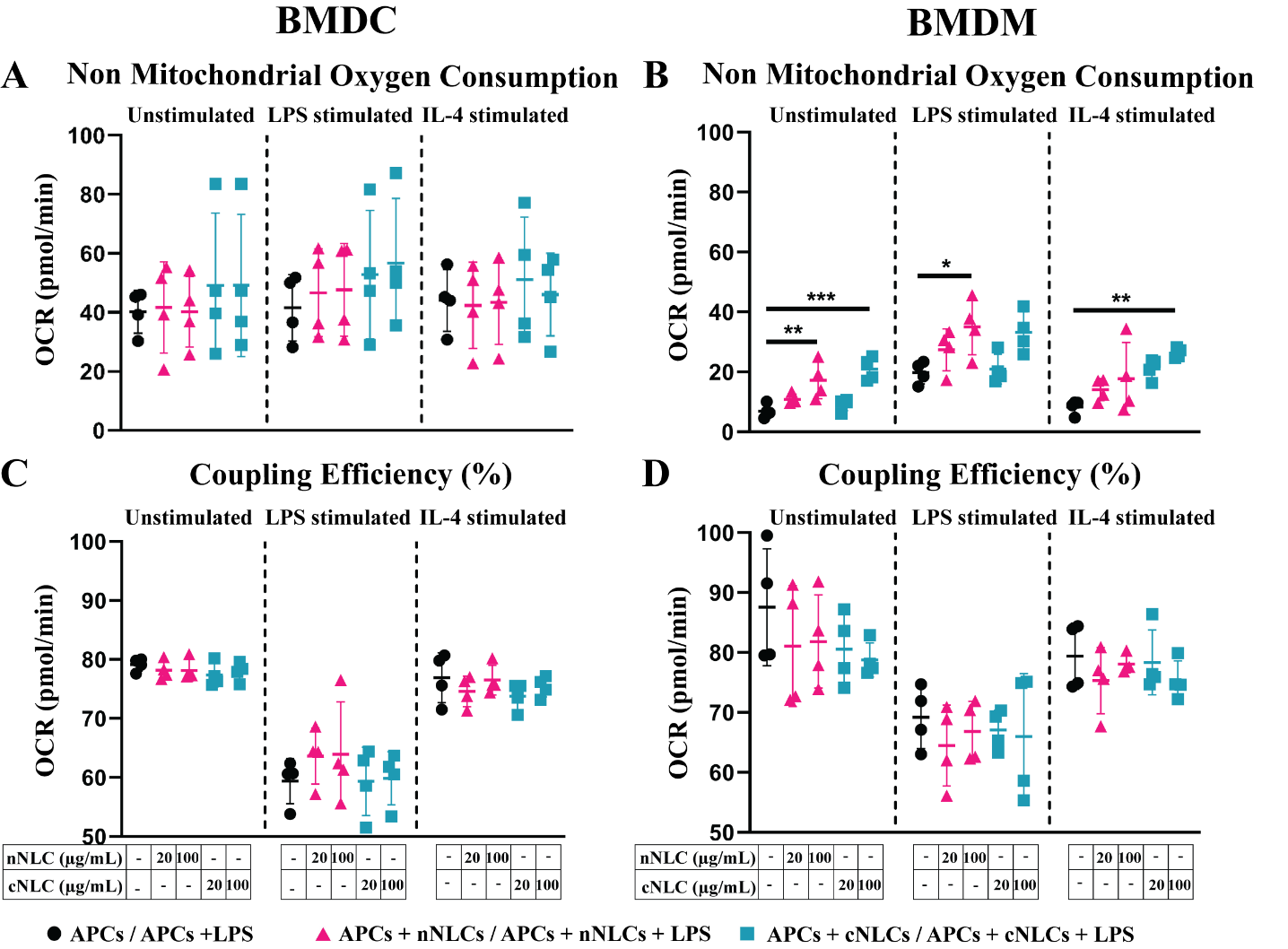


Supplementary Figure 6 | Non-mitochondrial oxygen consumption and percentage of coupling efficiency of naïve, classically activated or alternatively activated APCs in response to nNLCs or cNLCs. **(A, B)** Non-mitochondrial oxygen consumption, **(C, D)** percentage of coupling efficiency of BMDCs and BMDMs, respectively, were measured after exposure to cNLCs or nNLCs for 24 h and activated by LPS or IL-4 for another 24 h. Oxygen consumption rate (OCR) was quantified using a Seahorse XF analyser. Data was normalized by cell number based on cell count (Hoechst 33342 staining) and is displayed as mean ± SD (N = 4 independent experiments). Statistical significance between nanocarrier treated or untreated groups was performed by one-way ANOVA test using Tukey's multiple comparisons test. *P ≤ 0.05; **P ≤ 0.01; ***P ≤ 0.001; ****P ≤ 0.0001.

## Supplementary Figures 7.


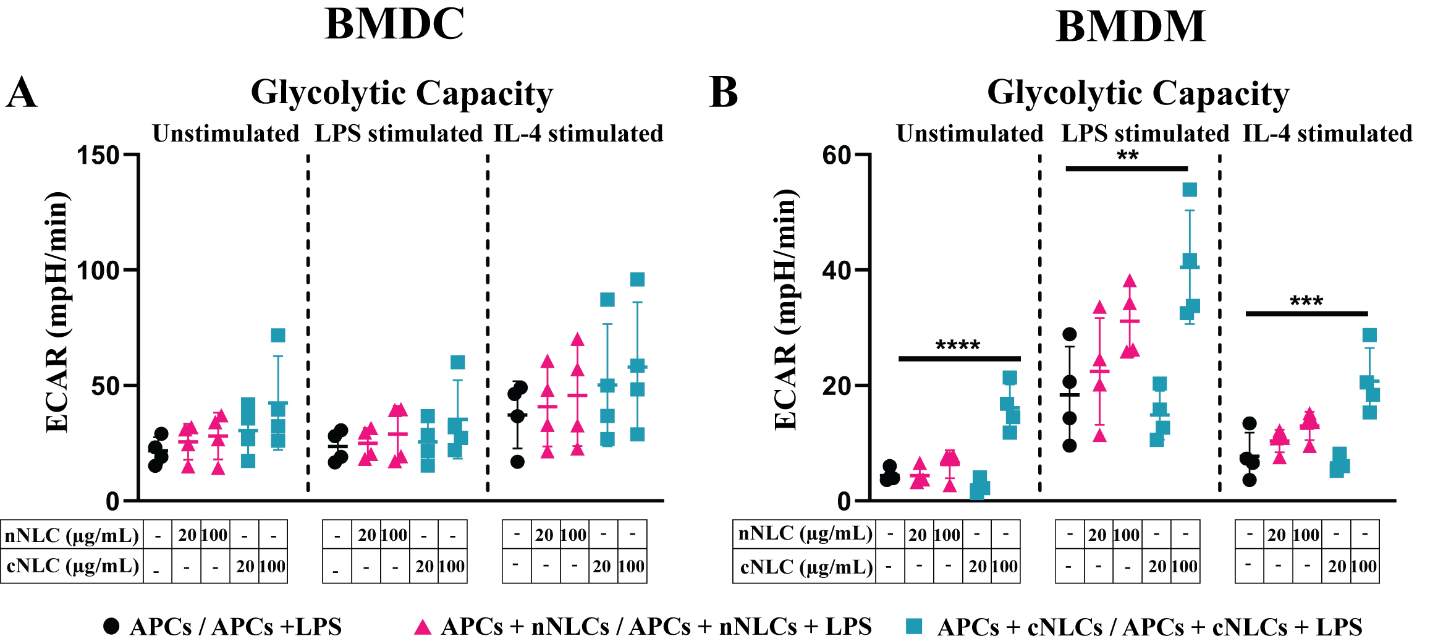


Supplementary Figure 7 | Glycolytic capacity of naïve, naïve, classically activated or alternatively activated APCs in response to nNLCs or cNLCs. Glycolytic capacity **(A)** in BMDCs and **(B)** in BMDMs were evaluated after exposure to cNLCs or nNLCs for 24 h and activated by LPS or IL-4 for another 24 h. Extracellular acidification rate (ECAR) was quantified using a Seahorse XF analyser. Data was normalized by cell number based on cell count (Hoechst 33342 staining) and is displayed as mean ± SD (N = 4 independent experiments). Statistical significance between nanocarrier treated or untreated groups was performed by one-way ANOVA test using Tukey's multiple comparisons test. **P ≤ 0.01; ***P ≤ 0.001; ****P ≤ 0.0001.

## Supplementary Figures 8.


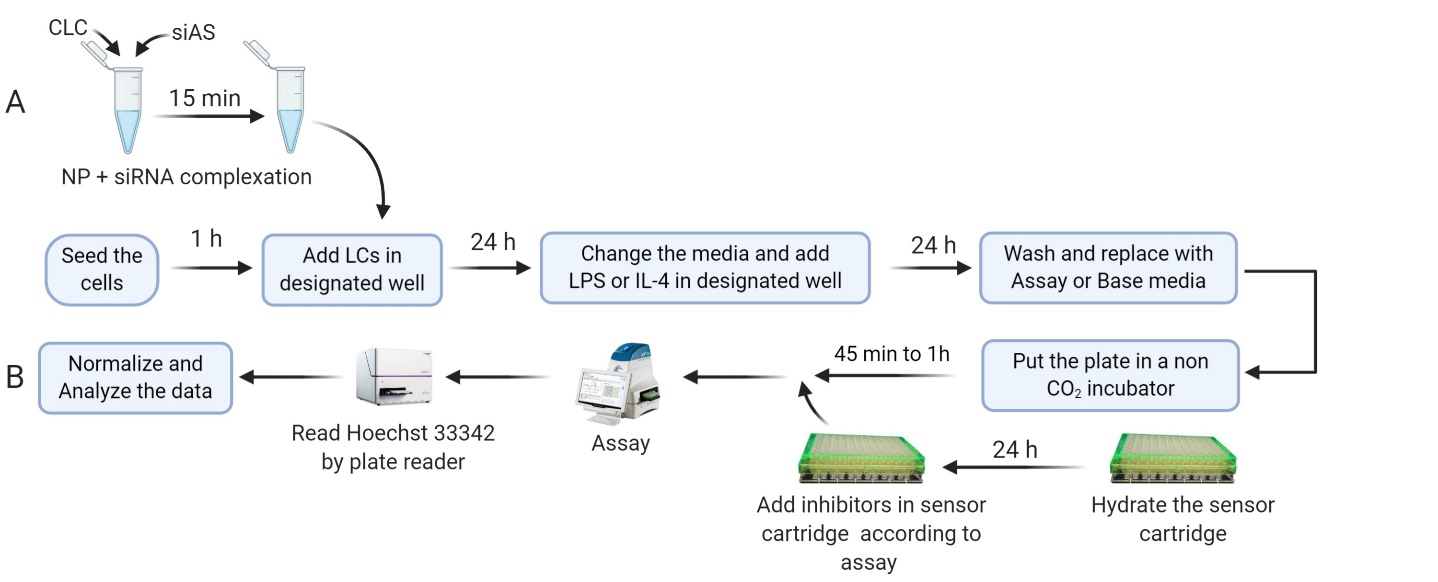


Supplementary Figure 8 | Experimental design of metabolic flux analysis for reversal of nanocarrier surface charge. Mature BMDCs and BMDMs were seeded in Seahorse culture plate. One hour after plating, cells were treated with the different nanocarriers, and when indicated with nanocarriers/siRNA nanocomplexes at the corresponding N/P ratios. After 24 h of culture, cells were washed and when indicated stimulated with LPS or IL-4 for 24 h. The metabolic analysis was performed using a Seahorse bio-analyser using the Mito Stress and Glyco Stress assay protocol, with the corresponding chemical inhibitors.

## Supplementary Figures 9.


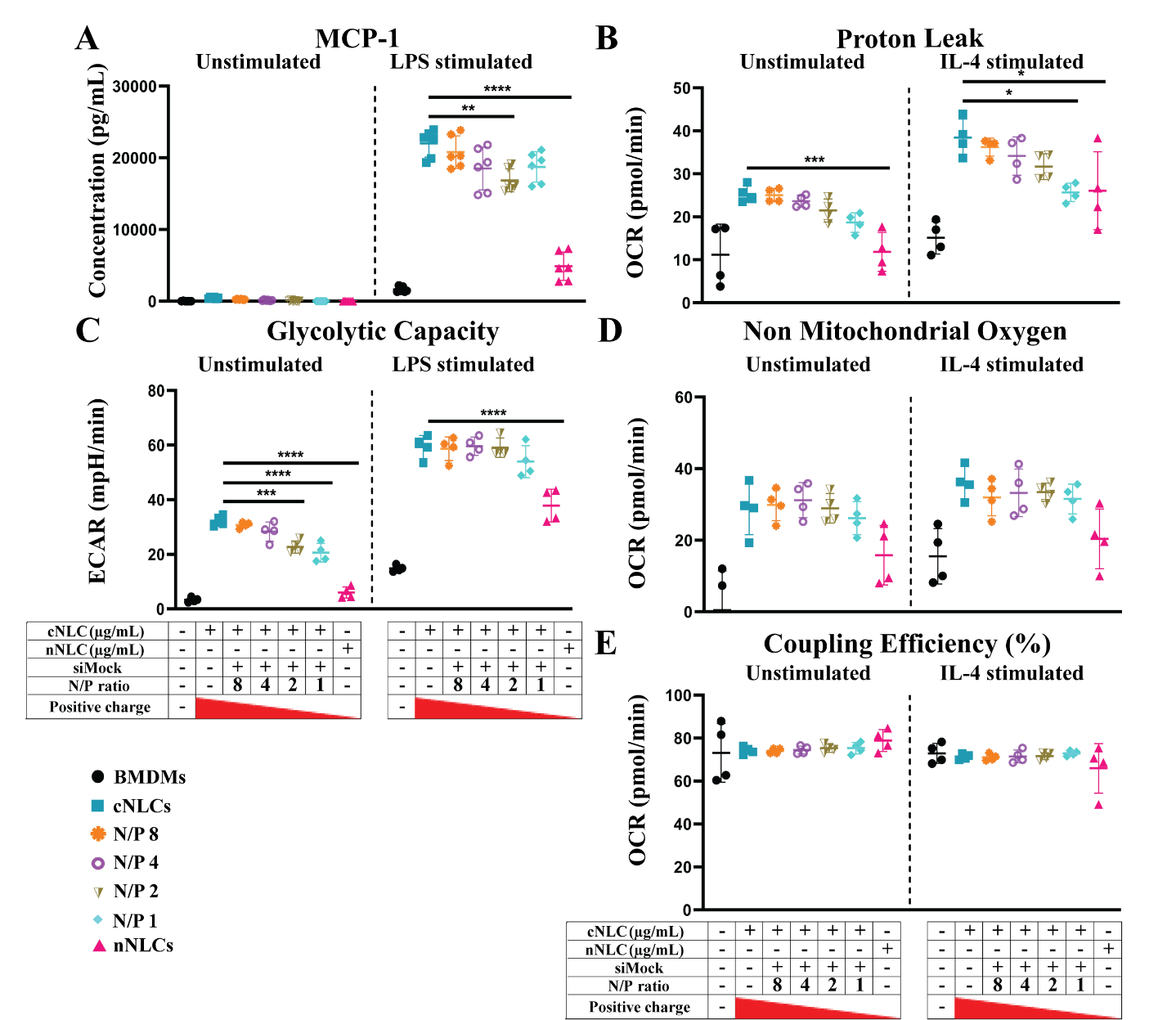


Supplementary Figure 9 | Effect of the net surface charge of cNLCs on different cellular functions and metabolism of BMDMs

**(A)** MCP-1 production was quantified from the supernatant of BMDMs exposed to 100 µg/mL of cNLCs complexes with siRNA at different N/P ratios and activated or not by LPS. **(B)** Glycolytic capacity in BMDMs exposed to 100 µg/mL of cNLCs complexes with siRNA at different N/P ratios and activated or not by LPS was determined by extracellular acidification rate (ECAR). **(C)** Proton leak **(D)** non-mitochondrial oxygen consumption, **(E)** percentage of coupling efficiency in BMDMs exposed to 100 µg/mL of cNLCs complexes with siRNA at different N/P ratios and activated or not by IL-4 was determined by Oxygen consumption rate (OCR). OCR and ECAR were quantified using a Seahorse XF analyser. Data was normalized by cell number based on cell count (Hoechst 33342 staining) and is displayed as mean ± SD (N = 3 independent experiments). Statistical significance between nanocarrier treated or untreated groups was performed by one-way ANOVA test using Tukey's multiple comparisons test. *P ≤ 0.05; **P ≤ 0.01; ***P ≤ 0.001; ****P ≤ 0.0001.
